# How shifting visual perspective during autobiographical memory retrieval influences emotion: A change in retrieval orientation

**DOI:** 10.3389/fnhum.2022.928583

**Published:** 2022-09-26

**Authors:** Selen Küçüktaş, Peggy L. St Jacques

**Affiliations:** Department of Psychology, University of Alberta, Edmonton, AB, Canada

**Keywords:** autobiographical memory (AM), visual perspective, emotion regulation, retrieval orientation, cognitive reappraisal, basic emotions, self-conscious emotions, functional neuroimaging (fMRI)

## Abstract

Visual perspective during autobiographical memory (AM) retrieval influences how people remember the emotional aspects of memories. Prior research in emotion regulation has also shown that shifting from an own eyes to an observer-like perspective is an efficient way of regulating the affect elicited by emotional AMs. However, the impact of shifting visual perspective is also dependent on the nature of the emotion associated with the event. The current review synthesizes behavioral and functional neuroimaging findings from the event memory and emotion regulation literature that examine how adopting particular visual perspectives and actively shifting across them during retrieval alters emotional experience, by primarily focusing on emotional intensity. We review current theories explaining why shifts in perspectives may or may not change the emotional characteristics of memories, then propose a new theory, suggesting that the own eyes and observer-like perspectives are two different retrieval orientations supported by differential neural activations that lead episodic details to be reconstructed in specific ways.

## Introduction

Autobiographical memories (AM) are often remembered with strong emotional reactions, particularly when emotional events are elicited. Depending upon the emotional nature of the remembered event, AMs can lead us to experience either positive (e.g., remembering a fun birthday party) or negative (e.g., remembering a severe injury) affective states (Holland and Kensinger, 2010). Although retrieving AMs may trigger intense emotional reactions, we are able to control our emotional responses and regulate them to alter their intensity and valence (Gross, 1998a,^2014^). One way of changing the emotional impact of AMs is by shifting visual perspective during retrieval, which is also considered as one of the most effective cognitive reappraisal strategies in emotional regulation research (McRae et al., 2012; Webb et al., 2012; Wallace-Hadrill and Kamboj, 2016). That is, visual perspective involves a cognitive change that alters how people experience emotions (Gross, 1998b; Ochsner and Gross, 2008). People can retrieve their AMs either from an own eyes perspective, visualizing events from where they were originally located while experiencing the event, or from an observer-like perspective, visualizing from an external point of view (Nigro and Neisser, 1983). Although own eyes perspectives are considered the dominant imagery perspective in AMs (Radvansky and Svob, 2019), most people can also flexibly shift to an observer-like perspective during retrieval (Robinson and Swanson, 1993; Rice and Rubin, 2009). Previous research has shown that shifting from an own eyes to an observer-like perspective during retrieval reduces subjective reports of emotional intensity during memory retrieval (e.g., Berntsen and Rubin, 2006; St Jacques et al., 2017; Marcotti and St Jacques, 2021). However, some theoretical models propose that shifting from an own eyes to an observer-like perspective might instead have no effect or even increase emotional reactions in some contexts (Sutin and Robins, 2008; Trope and Liberman, 2010; Libby and Eibach, 2011). In contrast, cognitive reappraisal research suggests that adopting an impartial observer’s perspective while pursuing an emotion regulation goal decreases negative emotion for various events (e.g., Ochsner et al., 2012; Buhle et al., 2014; Kross and Ayduk, 2017). Here, we review research on how shifting visual perspective influences the emotional aspects of AMs by including findings from event memory and cognitive reappraisal studies. We will first give an overview of the main theoretical models proposed to explain why shifting to an observer-like perspective influences the emotional aspects of the AMs. Then we describe evidence regarding how shifting perspective influences emotional intensity in AMs which is the particular focus of the current review, as well as the role of emotional valence and discrete emotional categories when there is a goal to regulate emotions or not. We will next highlight the brain mechanisms supporting how shifts in perspective during retrieval impact emotional aspects of memory. We will summarize the findings by presenting a new theory to explain why ivsual perspective impacts emotions and other characteristics of AMs based on retrieval orientation, and end with a discussion of the implications of this research and future directions.

## Theoretical explanations of the role of visual perspective on emotion in AM

Four main theories have been proposed to explain why adopting a particular visual perspective or shifting across multiple viewpoints influences emotional aspects of AM retrieval (see Table 1). Some theories suggest that visual perspective impacts emotional aspects of AM by altering the appraisal processes people engage in during memory retrieval (Wallace-Hadrill and Kamboj, 2016), while others suggest that shifting perspective influences emotional experiences by increasing psychological distance and the level of abstraction people engage in during memory retrieval (Moran and Eyal, 2022).

**TABLE 1:** Variables proposed to explain the impact of an observer-like perspective on emotion.

Variables	Self-Processes Model	Social-Cognitive Model	Construal Level Theory	Self-Reflection Model	Retrieval Orientation
An error in the conversion from LaTeX to XML has occurred here. 5*Evaluation of self-related An error in the conversion from LaTeX to XML has occurred here. 5*information	**↓** Emotion for the AMs incongruent with the self-concept	**—**	**—**	**—**	**—**
	**↑** Emotion for the AMs congruent with the self-concept				
Visibility of self	**↑** Emotion	**—**	**—**	**—**	**—**
An error in the conversion from LaTeX to XML has occurred here. 4*Meaning making	**—**	**↓** Emotion for abstract appraisal	**—**	An error in the conversion from LaTeX to XML has occurred here. 3*↓ Emotion via An error in the conversion from LaTeX to XML has occurred here. 3*reconstruing	**—**
		**↑** Emotion for concrete appraisal			
Psychological distancing	**—**	**—**	**↓** Emotion in a higher construal level	↓ Emotion by detaching from the event	**—**
An error in the conversion from LaTeX to XML has occurred here. 3*The nature of emotion	**↓** Basic emotions	**↓** Emotions leading to abstract appraisals	**↓** Emotions with lower construal level	**—**	**—**
	**↑** Self-conscious emotions	**↑** Emotions leading to concrete appraisals	**↑** Emotions with higher construal level		
Differential retrieval processes	**—**	**—**	**—**	**—**	The visual perspective cue orients the retrieval to decrease or increase emotion.

A dash represents that the given variable is not emphasized by the particular model. A downwards (upwards) arrow indicates a decrease (an increase) in emotional experience due to adopting an observer-like perspective.

**FIGURE 1:**
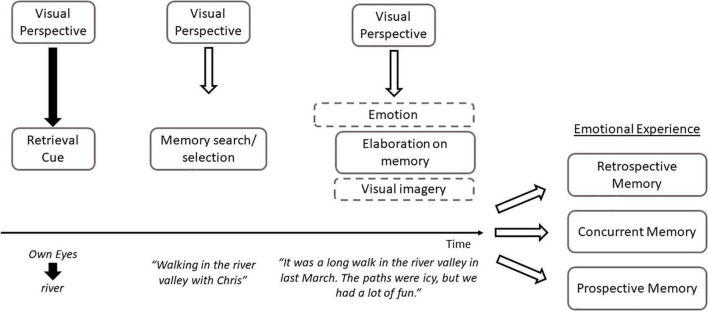
The impact of visual perspective shifts on emotional experiences during AM retrieval. Specifically, the model emphasizes that the presentation of a visual perspective cue (own eyes/observer) influences how the retrieval cue is processed, which biases later stages of retrieval, namely, the memory search/selection process and elaboration on memory details. Shifting to a novel perspective influences emotional experiences attached to the encoding context (retrospective memory), during retrieval (concurrent memory), and during subsequent retrieval (prospective memory).

If we consider shifts in visual perspective as an exclusive emotion regulation sub-strategy in the process model of emotion regulation (Ochsner and Gross, 2008), it could serve to alter the emotional impact of the event via cognitive change since people focus on the “internal environment” that provokes the emotional experience (e.g., memories, thoughts; Gross, 1998a,^2014^, ^2015^). Apart from this, some theories have suggested that one function of AM retrieval is to regulate emotions (e.g., Pasupathi, 2003; Holland and Kensinger, 2010; Harris et al., 2014). Explicit emotion regulation goals can influence which AMs are more accessible (e.g., recalling positive AMs to up-regulate emotions when feeling down) and how they are remembered (Pascuzzi and Smorti, 2017). The qualitative features of AMs (e.g., spontaneous own eyes and observer perspectives) emerge due to the natural characteristics of those memories. Then, manipulating these AM characteristics, such as shifting from an own eyes to an observer-like perspective, can impact various aspects of the memory (e.g., decreasing emotional intensity) and, thus, lead to an emotional regulation outcome. This does not need to be a controlled and effortful process; instead, it aligns with the idea that people can regulate their emotions automatically, without conscious effort, while thinking about the emotion-provoking event (Mauss et al., 2007; see Figure 1). Thus, we acknowledge that the theories reviewed below do not always scrutinize the effortful attempt of emotional regulation, as opposed to the process model of emotion regulation.

### Self-processes model

The self-processes model (Sutin and Robins, 2008) proposes that visual perspective can attenuate or amplify emotions depending upon how people evaluate self-relevant information during AM retrieval. Relying on the Self-Memory System (Conway and Pleydell-Pearce, 2000), this model argues that during retrieval the content of AM is evaluated in terms of its congruency and consistency with the self. Adopting a particular visual perspective then impacts how these self-evaluative processes alter the experience of subjective emotionality of the event. Sutin and Robins (2008) proposed two competing views to explain how this process occurs. First, the Dispassionate Observer view suggests that if an AM is incongruent with the self-concept or triggers a negative feeling, then adopting an observer-like perspective leads to an objective evaluation of events that reduces the affective feeling linked to the AM (e.g., Nigro and Neisser, 1983; Robinson and Swanson, 1993). Second, the Salient Self view suggests if an AM is congruent with the self-concept or elicits a positive feeling, then adopting an observer-like perspective amplifies emotional experiences associated with an AM by enhancing self-focused attention and making the self-relevant information more *salient*. Supporting this idea, Kinley et al. (2021) recently showed that the visibility of the self in an observer-like perspective is linked to the emotional intensity of future episodic thoughts. Specifically, when the self becomes more visible or *salient* during mental imagery, the experience of the emotional aspects of the event is heightened. Consequently, in both views, adopting an observer-like perspective entails a retrieval process that dampens or boosts the emotional experience as a result of engaging in a self-related evaluation about the memory content. The self-processes model also hypothesizes that the nature of the emotion linked to the memory influences the impact of the Dispassionate Observer and Salient Self views. In particular, adopting an observer perspective when retrieving AMs associated with self-conscious emotions (e.g., shame, pride) focuses attention on the self; thus, invokes a stronger self-evaluative process relative to basic emotions (e.g., sadness, happiness), which can amplify emotion for the former (e.g., Tracy and Robins, 2007a).

### Social-cognitive model

The social-cognitive model (Libby and Eibach, 2011; Niese et al., 2021) proposes that visual perspective leads to different processing styles in appraising events. In particular, adopting an own eyes perspective leads people to reflect on the concrete details of events (i.e., sensory-perceptual information), whereas adopting an observer-like perspective leads to greater reflection on the abstract or contextualized details of the memory. According to this model, adopting an observer-like perspective reduces the emotions related to the event by enabling people to detach from sensory-perceptual details in order to consider the event in a more abstract way. However, in some circumstances, adopting an observer-like perspective might lead people to think about the broader meaning of the memory in their lives. That is, if thinking about the broader meaning of an event to one’s life reduces (or *increases*) the emotional impact of the memory, then adopting an observer-like perspective also decreases (or *increases*) the emotional experiences. For example, Valenti et al. (2011) examined the impact of adopting an observer-like perspective on the feeling of regret. They found that adopting an observer-like compared to an own eyes perspective enhanced emotion for memories in which participants felt regret due to inaction, but diminished emotion for the memories in which participants felt regret due to their actions. Valenti and colleagues suggested that adopting an observer-like perspective increases the propensity to reflect on how regret for inactions fit into the broader meaning of one’s life, thereby boosting the emotions associated with these events.

### Construal level theory

Construal Level Theory (Trope and Liberman, 2010) proposes that people experience the “here and now” from an egocentric reference point, but can also engage in a process of psychological distancing by representing events at a spatiotemporally distant point in relation to the self. Psychological distancing in Construal Level Theory does not specifically refer to the shifts in visual perspective, but instead considers visual perspective as a component of social distancing where an event could be represented from an egocentric point-of-view or from the perspective of an external observer (Tausen et al., 2020). According to Construal Level Theory, adopting an observer-like perspective leads events to be construed in a more abstract and psychologically distanced manner. This distancing results in appraising events and objects with a higher mental construal that corresponds to a more abstract representation of the event; thus, attenuating the emotional intensity of remembering. Similarly, other theories suggest that adopting an observer-like perspective regulates emotion through psychological distancing (Powers and LaBar, 2019). Supporting these ideas, a number of studies have demonstrated that adopting an observer-like perspective increases subjective ratings of psychological distancing (e.g., Pronin and Ross, 2006; Van Boven et al., 2010; Gu and Tse, 2016). The nature of the emotion elicited can also interact with how visual perspectives influences psychological distance. For example, emotions that lead people to contemplate what other agents might think about them, such as shame or guilt, are linked to a higher construal level. In contrast, emotions such as anger or sadness do not require considering another agent’s perspective; thus, they are associated with a lower construal level (Trope and Liberman, 2010). A recent meta-analysis examining psychological distance and emotional experiences showed that psychological distancing had stronger effects on low-level than high-level emotions, such that adopting an observer-like perspective might amplify emotional experiences for emotions that involve a higher level of construal (e.g., guilt, shame), in contrast to emotions that involve a lower level of construal (e.g., sadness, anger; Moran and Eyal, 2022). Additionally, a specific emotional category might have a higher or lower level of construal depending on whether people focus on more abstract versus concrete features of the event during retrieval (e.g., Valenti et al., 2011; Doré et al., 2015).

### Self-reflection model

The self-reflection model (Kross and Ayduk, 2017) proposes that visual perspective influences whether people reflect on their feelings in an adaptive or maladaptive way. This model suggests that adopting an own eyes or *self-immersed* perspective leads people to focus more on what happened to them and how they felt, which induces people to engage in a ruminative process that intensifies the emotional impact of the event (Nolen-Hoeksema et al., 2008) and can be maladaptive when involving more negative experiences. In contrast, adopting an observer-like perspective or *self-distancing*, allows people to psychologically remove themselves from the event to interpret it more objectively and make sense of the experience, which diminishes emotions. The self-reflection model resembles Construal Level Theory, in highlighting the role of psychological distance, as well as the social-cognitive model, by emphasizing meaning-making when adopting an observer-like perspective. However, it is unique in its approach of examining how visual perspective influences recounting and reconstruing aspects of thought during AM retrieval (e.g., Kross et al., 2005; Kross and Ayduk, 2008). For example, Kross and Ayduk (2008) asked participants to describe their thought contents while retrieving a sad and depressive AM from an own eyes or an observer-like perspective. They found that own eyes perspectives were associated with greater recounting (focusing more on what happened and how felt; e.g., “I went to the top of the stairwell and cried for a long time”), which led to a greater emotional response during retrieval. In contrast, adopting an observer-like perspective was associated with greater reconstruing (psychologically removing from the event to interpret it more objectively and make sense of the experience; e.g., “I thought about how foolish it seems in retrospect”), and less emotional experience during retrieval.

Taken together, the proposed models have different emphases regarding how visual perspective impacts emotional experience. The self-processes model mainly focuses on the role of self-evaluation when adopting an observer-like perspective in which people interpret the congruency of an AM with their self-concept. The social-cognitive model proposes that alternative visual perspectives lead to concrete versus abstract ways of thinking about the event during retrieval. Construal Level Theory considers observer-like perspective as a particular example of psychological distancing that leads events to be recalled with a higher construal level. Finally, the self-reflection model highlights the processes people engage in to make sense of their feelings by adopting a particular visual perspective. Additionally, the first three models emphasize that the impact of adopting an alternative visual perspective depends upon the nature of the emotion associated with the event, and the last model underlines how memory content specifically changes due to visual perspective.

## The impact of shifting visual perspective on the emotional intensity of memories

Evidence from event memory research has revealed that the link between visual perspective and the emotional intensity of memories is bidirectional (Rice, 2010). On the one hand, the emotional intensity of an event influences the visual perspective that people spontaneously adopt during retrieval (Nigro and Neisser, 1983). For example, emotional events are more likely to be recalled from an own eyes than an observer perspective (e.g., D’Argembeau et al., 2003; Talarico et al., 2004; but see Libby and Eibach, 2011). On the other hand, the visual perspective adopted during retrieval can also alter how we experience the emotional intensity of memories, such that memories associated with own eyes perspectives are higher in emotional intensity than memories associated with observer perspectives (e.g., McIsaac and Eich, 2002). In this section, we review findings that reveal how spontaneously adopting an own eyes or observer perspective and shifts in perspective influence the emotional intensity of memories.

The viewpoint that people naturally adopt when recalling memories influences the emotional intensity they experience during retrieval (e.g., Nigro and Neisser, 1983; Berntsen and Rubin, 2006). In their seminal study, Nigro and Neisser (1983) instructed participants to recall AMs and then select the visual perspective they naturally adopted among dichotomous options and to provide subjective ratings of emotional intensity. They found that AMs naturally retrieved from an own eyes compared to an observer-like perspective were higher in emotional intensity. Later studies confirmed that people are more likely to naturally adopt an own eyes rather than an observer-like perspective during the retrieval of emotional events (e.g., D’Argembeau et al., 2003; Talarico et al., 2004). Other research has shown that adopting an own eyes perspective led to an increase in the emotional intensity and affective details in memory descriptions for lab-based mini-events and fictional stories (McIsaac and Eich, 2002; Bagri and Jones, 2009; Eich et al., 2009), suggesting that the relationship between viewpoint and emotion extends to other types of event memories irrespective of their personal relevance or emotional significance. A few studies have also shown that visual perspective not only impacts subjective feeling but can also cause physiological measures of emotional arousal, such that adopting an observer-liker perspective is associated with less cardiovascular (Ray et al., 2008) and blood pressure reactivity (Ayduk and Kross, 2008). These findings indicate that self-reported reductions in emotional intensity when adopting an observer-like perspective are also evident by parallel changes in objective emotional experience. Given that remote memories are more likely to be recalled from an observer-like perspective and with reduced emotional experience than recent memories (e.g., Talarico et al., 2004; Sutin and Robins, 2007; Rice and Rubin, 2009), a critical question is whether similar mnemonic changes in emotion occur when visual perspective is manipulated during retrieval.

Several studies have shown that shifting from an own eyes to an observer-like perspective influences emotional intensity (e.g., Robinson and Swanson, 1993; Berntsen and Rubin, 2006; St Jacques et al., 2017). For example, St Jacques et al. (2017) investigated how shifting from a dominant own eyes to an alternative observer-like perspective influenced subjective reports of emotional intensity during retrieval. Participants were asked to generate AMs from their natural visual perspective and then rate visual perspective and emotional intensity. The experimenters then selected a subset of memories strongly associated with a natural own eyes perspective based on the participant ratings. In Session 2, one week later, the retrieval of these memories was directly manipulated by asking participants to either maintain the same own eyes perspective or shift to an alternative observer-like perspective. St. Jacques et al. found that shifting from a dominant own eyes to an alternative observer-like perspective during retrieval decreased the emotional intensity of AMs, compared to maintaining a dominant own eyes perspective. Similarly, some studies have shown that shifting from an own eyes to an observer-like perspective can also reduce the emotional valence of AMs (e.g., Vella and Moulds, 2014; Speed et al., 2020). Other research has shown that shifting perspective influences emotional aspects of how memories are described (Crawley, 2010; Gu and Tse, 2016; Akhtar et al., 2017; King et al., 2022). For example, Akhtar et al. (2017) asked participants to retrieve AMs from their natural perspective and then shift to the opposite visual perspective while providing a narrative describing their memory. They found that emotional intensity was reduced when shifting from an own eyes to an observer-like perspective and that participants also described their memories using fewer affective details. Similarly, Gu and Tse (2016) asked participants to provide narrative descriptions of emotional AMs, while either shifting from first-person to third-person pronouns or vice versa. They found that a shifting in writing AMs from first-person to third-person pronouns reduced subjective ratings of emotional intensity. Importantly, the direction of the shift predicted the changes in psychological distance ratings such that shifting from first-person to third-person pronouns was associated with increased psychological distance, which also mediated the effect of shifting from first- to third-person pronouns on emotional intensity. Adopting an observer-like perspective during memory retrieval can also influence retrospective reports of the emotions people thought they experienced during memory encoding. For example, Crawley (2010) asked participants to rate their remembered emotional intensity experienced at the time of the event following a shift from an own eyes to an observer perspective during AM recall and found a reduction in the remembered emotional intensity across repeated retrievals. Taken together, prior research indicates that manipulating visual perspective influences multiple aspects of the emotional experience of remembering including the emotional intensity experienced during retrieval, the affective information used to describe narrative of these events, and how people remember the emotional intensity attached to the original event.

Only a couple of studies, to our knowledge, have examined whether the proximate effects of shifting perspective on emotional experience during remembering impact how memories are later recalled from their natural perspective (Sekiguchi and Nonaka, 2014; King et al., 2022). In one study, Sekiguchi and Nonaka (2014) examined whether the proximate reductions in emotional intensity persisted when memories were tested a few weeks after the visual perspective manipulation. In Session 1, they asked participants to retrieve emotional AMs from their natural visual perspective and rate emotional intensity. In Session 2, a few days later, participants were asked to shift to the opposite perspective of what they naturally adopted in Session 1. A final memory test took place a few weeks later, in which participants recalled the same events from their natural visual perspective and rated emotional intensity. The results showed that shifting to an observer perspective caused a reduction in the emotional intensity during Session 2, and that these effects persisted even when memories were retrieved from their natural perspective a few weeks later. In another study, King et al. (2022) found a similar reduction in emotional intensity as the result of shifting from an own eyes to an observer-like perspective when memories were recalled from their natural point-of-view two days later. Additionally, this study also examined how shifting perspective influenced the emotion/thoughts participants used when describing autobiographical narratives. They found proximate effects of shifting from an own eyes to an observer-like perspective on emotion/thoughts, as reflected by a reduction in these details compared to the original narratives. However, these changes in emotion/thought details did not persist during later recall of the same memories from their natural point-of-view. Although participants reported less subjective feeling in memories in which they had previously shifted to an observer perspective, there were no changes in the amount of emotion/thought details they provided in their narratives. This disassociation between subjective and objective measures of emotionality suggests that shifting to an observer-like perspective might impact how people re-experience the subjective emotional intensity, but not objective changes in how these events are described. Similarly, other research has shown that retrieving AMs from a different perspective than how they were initially recalled can lead to long-lasting changes in other characteristics of memories, such as subjective vividness and the natural viewpoint adopted (Butler et al., 2016; St Jacques et al., 2017), as well as the accuracy of memories (Marcotti and St Jacques, 2018, 2021).

A consistent finding in the literature is that the changes in emotional intensity due to shifting perspectives occur unidirectionally (Robinson and Swanson, 1993). While shifting from an own eyes to an observer-like perspective reduces the emotional intensity, there is not a similar *increase* when shifting in the reverse direction (e.g., Berntsen and Rubin, 2006; Sekiguchi and Nonaka, 2014; Vella and Moulds, 2014; Gu and Tse, 2016; Akhtar et al., 2017). Few studies, to our knowledge, have reported a lack of reduction in emotional intensity when shifting from an own eyes to an observer-like perspective (e.g., Marcotti and St Jacques, 2018; St Jacques et al., 2018). However, in these studies, participants engaged in non-emotional lab-based mini-events (Marcotti and St Jacques, 2018) or were explicitly instructed not to change the emotional aspects of the events in specific conditions (St Jacques et al., 2018). To explain the unidirectionality, some theories suggest that asymmetrical effects are due to the loss of experiential information when adopting an observer-like perspective, such that shifting to an own eyes viewpoint is not effective in recovering emotional information associated with the memory (Robinson and Swanson, 1993). Berntsen and Rubin (2006) proposed that increasing the recollective experiences during retrieval might be cognitively more demanding than decreasing them; thus, impending the ability to generate emotional aspects of remembering when shifting from an observer to an own eyes perspective. Other proposals suggest that repeated retrieval from an observer-like perspective leads to the loss of visual information over time, such that reinstating recollective experiences when shifting back to an own eyes perspective may not be possible (Butler et al., 2016). One potential issue with these ideas is that they assume that observer memories were originally encoded from an own eyes perspective, and then emerge as the result of shifting to an observer-like perspective during retrieval. Thus, shifting from an observer to own eyes perspective is not the same as shifting in the reverse direction, since in the former people are shifting back to the original point-of-view during encoding, whereas in the latter they are shifting to a novel perspective. Some theories argue that memories can also be encoded from an observer-like perspective (e.g., Nigro and Neisser, 1983; McCarroll, 2017, 2018), consistent with a growing number of studies have shown that it is possible to form memories from an observer-like perspective (Bergouignan et al., 2014; Mooren et al., 2016; Iriye and St Jacques, 2021). Examining shifts from observer to own eyes perspectives in memories originally formed from an observer-like perspective would help to better understand the pattern of asymmetrical effects on emotion. Moreover, shifting to a visual perspective that differs from perception during encoding of emotionally laden events would impact how the emotional aspects of the event will be formed in the memory (McCarroll, 2018). In other words, shifting across alternative visual perspectives during encoding can be beneficial by facilitating the down-regulation of the intensity of a negative emotion even before the event is completely formed in the memory.

In sum, the flexible nature of memories enables us to adopt alternative visual perspectives and actively shift across them during retrieval, which reduces subjective and objective measures of emotional experience in memories when shifting from an own eyes to an observer-like perspective. These mnemonic changes that occur due to shifting visual perspective are consistent with theory indicating that retrieval is an active process that can reshape and update memories (Hardt et al., 2010; Schacter et al., 2011; McDermott et al., 2016; St Jacques, 2019), which might have beneficial long-term impacts for well-being and mental health by modifying the emotional aspects of negative AMs as an adaptive emotional regulation strategy (Kross and Ayduk, 2008). Current evidence does not strongly favour existing theories of visual perspective. The reduction in emotional intensity in the studies in which emotional memories were not exclusively targeted draws into question whether the nature of the triggered emotion modulates the impact of shifting perspective on emotion as the self-processes model would predict. Likewise, instructing participants to watch themselves from an observer-like perspective, that possibly increases the visibility of the self, did not prevent the decrease in emotional intensity (e.g., Akhtar et al., 2017; St Jacques et al., 2017), as also predicted from this model. Only Gu and Tse (2016), supporting Construal Level Theory, have shown that the direction of shifting perspective predicted the ratings of psychological distance such that shifting from first-person to third-person pronouns was related to increased psychological distance. Therefore, alternative explanations are required to clarify why shifts in visual perspective influence emotional intensity.

## The impact of visual perspective on emotional valence and discrete emotional categories

The influence of visual perspective on memory might differ depending upon the nature of the emotions elicited. Emotions in AMs can be categorized based on their valence (i.e., positive, negative, or neutral; Russell and Carroll, 1999) or whether they involve discrete emotional categories (e.g., sadness, shame; Tracy and Robins, 2007a). In particular, a number of studies have focused on the impact of visual perspective during AM retrieval on emotional experiences that rely on self-evaluative processes that elicit self-conscious and basic emotions (Tracy and Robins, 2007b). This section examines the effect of visual perspective during AM retrieval for emotional valence and discrete emotional categories.

Prior research has revealed inconsistent findings regarding the relationship between visual perspective and emotional valence (for review see Rice, 2010). Despite earlier findings suggesting that positive and negative events, relative to the neutral ones, are more likely to be recalled from an own eyes perspective (e.g., D’Argembeau et al., 2003), later studies showed that this relationship might not be as robust with some studies showing differences for negative but not positive valence (McFadden and Siedlecki, 2020) or failing to show any causal differences or an association between emotional valence and visual perspective (e.g., Berntsen and Rubin, 2006; Siedlecki, 2015). Similarly, studies manipulating visual perspective during AM retrieval have also not found differences in the impact of shifting perspective on positive versus negative AMs (Berntsen and Rubin, 2006). Research targeting more highly negative and stressful events have shown more robust effects of visual perspective, such that traumatic memories are frequently recalled from an observer-like perspective compared to positive and neutral memories (e.g., Porter and Birt, 2001; Berntsen et al., 2003; McIsaac and Eich, 2004; Kenny and Bryant, 2007). However, some of these effects might be due to the arousing nature of these events rather than their particular valence. Overall, the inconsistent relationship between emotional valence and visual perspective supports other research indicating that emotional valence is not as strong a determinant of the characteristics of AMs, including perspective, when compared to emotional intensity (e.g., Talarico et al., 2004).

Visual perspective does seem to have an impact on AM retrieval for events involving self-conscious versus basic emotions. For example, self-conscious emotions are associated with higher naturally occurring observer-like perspectives during AM retrieval (D’Argembeau and Van der Linden, 2008; but see Terry and Horton, 2007). Similarly, several studies have shown that manipulating visual perspective during retrieval differentially impacts self-conscious and basic emotions (e.g., Valenti et al., 2011; Hung and Mukhopadhyay, 2012; Katzir and Eyal, 2013; Cândea and Szentágotai-Tãtar, 2020). For example, Katzir and Eyal (2013) experimentally manipulated how adopting own eyes or observer-like perspectives during retrieval of self-conscious (i.e., guilt, shame) and basic (i.e., anger, sadness) emotions. They found that adopting an observer-like compared to an own eyes perspective decreased the intensity of anger and sadness, but did not affect guilt and shame. Other research, however, has demonstrated that adopting an observer-like perspective can amplify self-conscious emotions in some contexts (e.g., Libby et al., 2011; Valenti et al., 2011; Hung and Mukhopadhyay, 2012; Krishnamoorthy et al., 2021; Moran and Eyal, 2022). For example, Hung and Mukhopadhyay (2012) showed that adopting an observer perspective when visualizing hypothetical events increased the intensity of self-conscious emotions, whereas adopting an own eyes perspective increased the intensity of hedonic based emotions related to the situation itself (e.g., joy, excitement). In fact, prior research indicates that adopting an observer-like perspective requires an additional emotion regulation goal in order to effectively reduce self-conscious emotions (Valenti et al., 2011; Hung and Mukhopadhyay, 2012; Katzir and Eyal, 2013; Powers and LaBar, 2019; Cândea and Szentágotai-Tãtar, 2020). For example, Krishnamoorthy et al. (2021) examined how adopting own eyes or observer-like perspectives when recalling AMs associated with shame influenced the intensity of feelings of shame in individuals who were categorized as high-shame or low-shame prone. They found that adopting an observer-like perspective compared to an own eyes perspective led to higher feelings of shame in the high-shame group, but there were no differences in feelings of shame due to perspective in the low-shame group. However, when the shift in perspective was combined with an emotional regulation goal to decrease emotion (through positive reappraisal), feelings of shame were also reduced in the high-shame group. Downregulating emotional experiences that elicit self-conscious emotions by adopting an observer perspective might be more challenging due to increased attention focused on the self that triggers negative self-evaluations (e.g., “I feel incapable”; Cândea and Szentágotai-Tãtar, 2020) or lead individuals to focus on how other people might think about them (e.g., “I saw she was disappointed in me”; Katzir and Eyal, 2013). Thus, in contrast to basic emotions, adopting an observer-like perspective might be ineffective in dampening self-conscious emotions due to salient negative self-evaluations. Overall, the evidence supports both the self-processes and social-cognitive models, regarding the differential effects of alternative visual perspectives depending on the nature of triggered emotion (e.g., Katzir and Eyal, 2013) and the appraisals that are possibly generated while thinking about the event (e.g., Valenti et al., 2011; Cândea and Szentágotai-Tãtar, 2020; Krishnamoorthy et al., 2021). These findings also raise the question of whether an explicit positive reappraisal is required for visual perspective shifts to serve as an emotion regulation strategy for certain types of events, which is important for understanding the impact of shifting perspective to regulate emotions in mental disorders such as social anxiety (Spurr and Stopa, 2003) and PTSD (McIsaac and Eich, 2004).

Taken together, prior research has not revealed a strong relationship between visual perspective and emotional valence. In contrast, visual perspective does differentially impact self-conscious and basic emotions. The research reviewed here indicates that adopting an observer-like perspective might reduce basic emotions, but amplify self-conscious emotions. Thus, for self-conscious emotions, adopting an observer-like perspective might only be an effective emotional regulation strategy when coupled with an emotional regulation goal. These findings also highlight the importance of isolating self-conscious from basic emotional cues when examining potential differences in the impact of visual perspective on emotional valence during AM retrieval, as blurring these different types of emotional experiences might contribute to inconsistencies in the literature.

## Neural mechanisms of shifting visual perspective on emotional intensity

AM retrieval is supported by neural recruitment in brain regions overlapping with the default and frontoparietal networks (Svoboda et al., 2006; Cabeza and St Jacques, 2007; Spreng et al., 2009), including regions in the medial and lateral temporal lobe, posterior parietal cortices, and medial and lateral prefrontal cortex (PFC). Visual perspective during AM retrieval is supported by neural recruitment of the precuneus and angular gyrus (St Jacques, 2022). Virtual lesions to either the precuneus or angular gyrus alter visual perspective during AM retrieval (e.g., Bonnici et al., 2018; Hebscher et al., 2020), and these regions are also recruited when participants are asked to shift from an own eyes to an observer-like visual perspective when compared to maintaining an own eyes perspective (St Jacques et al., 2017, 2018; Faul et al., 2020; Iriye and St Jacques, 2020). Emotional aspects of AM retrieval elicit additional activity in the amygdala (Fink et al., 1996; Markowitsch et al., 2000; Greenberg et al., 2005; Daselaar et al., 2008; Ford and Kensinger, 2019), which through its interactions with the hippocampus contribute to better remembering of emotional experiences (Holland and Kensinger, 2010). Functional neuroimaging studies of emotional regulation research have further revealed that lateral and medial PFC (e.g., Fabiansson et al., 2012; Holland and Kensinger, 2013; Doré et al., 2018; but see Kross et al., 2009), contribute to the down-regulation of emotional responses in the amygdala when regulating emotions during retrieval (Denkova et al., 2013, 2015; for a review see Dolcos et al., 2017). However, some studies have also implicated the role of the precuneus in emotional regulation of AMs (Holland and Kensinger, 2013; St Jacques et al., 2017; also see Dörfel et al., 2014 for non-AM stimuli) and have suggested that altering the visual imagery of AMs can serve to reduce emotional responses during remembering (e.g., Holland and Kensinger, 2010). In their neurocognitive model, Powers and LaBar (2019) proposed that the temporal parietal junction, which encompasses the angular gyrus, might further contribute to emotional regulation as the result of distancing through its role in perspective taking.

Only a handful of studies have directly examined the neural mechanisms by which shifting visual perspective impact emotional aspects of AM (Grol et al., 2017; St Jacques et al., 2017; Doré et al., 2018; also see Eich et al., 2009). In one fMRI study, St Jacques et al. (2017) asked participants to maintain an own eyes perspective or shift to an observer-like perspective during AM retrieval. They found greater neural recruitment in the precuneus, angular gyrus, and lateral PFC when shifting to an observer perspective. Additionally, reductions in emotional intensity ratings as the result of shifting perspective were predicted by neural recruitment of the precuneus, consistent with the suggestion that neural recruitment of visual imagery regions might also contribute to emotional regulation. Similarly, Grol et al. (2017) found greater recruitment of both precuneus and angular gyrus when adopting an observer compared to an own eyes perspective during recall of positive and neutral AMs. There were also no significant differences when shifting perspective in positive or neutral AMs, which dovetails with the behavioral research reviewed above. In another study, Doré et al. (2018) investigated how adopting a particular visual perspective while pursuing an emotion regulation goal impacts neural recruitment during AM retrieval. Participants were asked to retrieve negative AMs by adopting an own eyes perspective (visualizing the event as if they were immersed in it and letting their emotions unfold) or an observer-like perspective (visualizing the event from a distance and an external observer’s perspective focusing on the facts related to the event). They found that relative to an own eyes perspective, retrieving negative AMs from an observer-like perspective was associated with greater neural recruitment in posterior parietal cortices and dorsolateral PFC, coupled with less neural recruitment in both the amygdala and hippocampus. The behavioral findings further revealed that adopting an observer-like perspective reduced both negative affect and vividness, which is consistent with the idea that changes in visual imagery are related to similar changes in emotional experience during AM retrieval.

**FIGURE 2:**
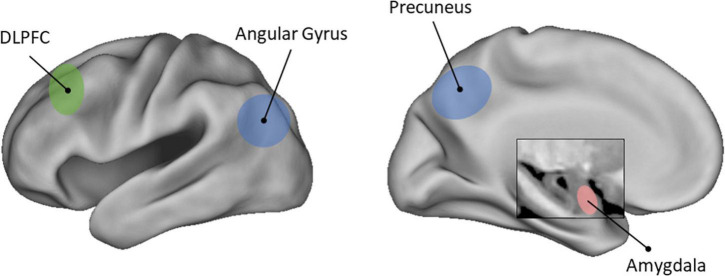
Brain regions that support changes in emotion due to shifting visual perspective during AM retrieval. Precuneus and angular gyrus (blue-colored) support the representation and updating of memories from a particular visual perspective. When emotional regulation goals are present, additional recruitment of dorsolateral prefrontal cortex (DLPFC; green-colored) helps to further and attenuate emotional arousal in the amygdala (red-colored).

In sum, shifting to a novel visual perspective is supported by the regions within the posterior parietal cortex, which might impact emotional aspects of AM retrieval by altering visual imagery during remembering (see Figure 2). Additional recruitment of PFC could further contribute to changes in emotional experience when adopting an observer perspective, and, when this shift in perspective is in the pursuit of an emotional regulation goal, dampen emotional responses in the amygdala (Doré et al., 2018). These findings also highlight that AMs can be remembered in multiple ways that serve different adaptive functions (e.g., Sheldon et al., 2019). Shifting to a novel perspective can lead to changes in perceptual aspects of remembering that alter emotion, as well as conceptual aspects of remembering, when the goal is to re-evaluate the emotional outcome of events from this new perspective.

## Discussion

Visual perspective in AM is closely linked to how people experience the emotional aspects of events during retrieval. Naturally adopting a particular visual perspective or actively shifting perspective influences both subjective and objective measures of emotionality. In particular, prior research shows that observer-like perspectives are frequently associated with a decreased emotional intensity when shifting from an own eyes to an observer-like perspective. However, the impact of shifting on emotionality is unidirectional, with no predicted increase when shifting from an observer-like to an own eyes perspective. Earlier theories proposed that the reduction in emotional intensity due to shifting perspective was linked to meaning-making about the event by reappraising it more objectively in an observer perspective (Libby and Eibach, 2011; Niese et al., 2021) or increasing psychological distance to a higher construal level (Trope and Liberman, 2010) which allows people to analyze their feelings more objectively to regulate their affect (Kross and Ayduk, 2017). While these findings seem to hold for basic emotions, a different pattern of effects is evident for self-conscious emotions, such that observer perspectives do not influence the self-conscious emotions or might even heighten them in some contexts (Sutin and Robins, 2008). Although only a few studies have examined the neural mechanisms by which visual perspective impacts emotional experience during AM remembering, this work demonstrates the involvement of the precuneus and angular gyrus in supporting the reduction in emotional intensity due to shifting from an own eyes to an observer-like perspective. Yet, there are remaining questions regarding the mechanisms by which shifts in visual perspective influence emotional aspects of memories.

Current theories suggest that the changes in emotional experience due to shifting perspective are linked to factors such as self-evaluative processes (Sutin and Robins, 2008), abstract versus concrete thinking while appraising the broader meaning of the event (Libby and Eibach, 2011; Niese et al., 2021), increased psychological distance (Trope and Liberman, 2010), and self-reflective processes (Kross and Ayduk, 2017). These theories have contributed to understanding why shifting visual perspective impacts emotional experiences, particularly when there are explicit emotion regulation goals (e.g., Krishnamoorthy et al., 2021), meaning-making (Valenti et al., 2011), or active consideration of negative self-evaluations (e.g., Hung and Mukhopadhyay, 2012; Cândea and Szentágotai-Tãtar, 2020). However, shifts in perspectives can alter emotional characteristics of events even when emotional AMs were not specifically targeted and there are no specific emotional regulation goals (e.g., Berntsen and Rubin, 2006; Sekiguchi and Nonaka, 2014; St Jacques et al., 2017; King et al., 2022). Moreover, prior theories do not consider episodic memory retrieval processes that might contribute to changes due to visual perspective during remembering. For example, as reviewed above, changes in visual imagery due to shifts in perspectives during retrieval might also contribute to changes in emotional aspects of AMs, but the critical role of visual imagery in AM has largely been neglected by prior theories of visual perspective in memory. Another important aspect of episodic retrieval that might contribute to changes in AM due to visual perspective is retrieval effort. For example, several studies have found that shifting from an own eyes to an observer perspective is more effortful than maintaining an own eyes perspective (St Jacques et al., 2017, 2018; Iriye and St Jacques, 2020). While differences in retrieval effort account might explain reported decreases in memory retrieval, it cannot easily account for increases in memory retrieval due to shifting perspective (e.g., King et al., 2022). Nonetheless, additional research could aim to better control for these differences in retrieval demands when comparing different visual perspective conditions (e.g., Iriye and St Jacques, 2021).

Here, we propose that own eyes and observer-like perspectives represent two distinct retrieval orientations during AM retrieval that bias emotional and other recollective aspects of remembering. Retrieval orientation refers to differences in how retrieval cues are processed and can impact the effectiveness of memory retrieval depending upon whether this processing overlaps with similar processes engaged during memory encoding (Rugg and Wilding, 2000; Herron and Rugg, 2003). Prior research has shown that changes in how retrieval cues are processed can bias neural activity prior to and during episodic memory retrieval (e.g., Herron and Rugg, 2003; Hornberger et al., 2006; Morcom and Rugg, 2012). Recent research has also shown that retrieval orientation can lead to similar biases in AM retrieval by influencing the underlying brain networks that contribute to remembering (Gurguryan and Sheldon, 2019), and has linked these retrieval orientations to different functions of AM remembering (Sheldon et al., 2019). Similarly, adopting an own eyes or observer-like perspective also influence how underlying memory representations are prioritized during AM retrieval. For example, in an fMRI study Iriye and St Jacques (2020) demonstrated that adopting a particular perspective biased pre-retrieval processes that guided how particular AMs were initially constructed and later elaborated upon. Participants were asked to retrieve AMs cued by familiar spatial locations while adopting own eyes and observer-like perspectives. They found that when participants were cued to adopt an observer-like perspective during AM retrieval there was greater functional connectivity between the hippocampus and posterior parietal cortices during a pre-retrieval phase, when participants were asked to search for and select a particular AM. Additionally, adopting an observer-like perspective was also associated with less engagement of the AM retrieval network once a particular memory was recovered and participants were asked to elaborate upon retrieval of the memory in as much details as possible. Thus, adopting a particular perspective influenced pre-retrieval processes and contributed to the effectiveness of memory retrieval (Hebscher et al., 2020). In other words, the impact of adopting a particular visual perspective on memory could be determined starting from the early phases of AM retrieval-even before later retrieval stages in which people would engage in complex self-evaluative or meaning-making processes, as suggested by prior theories.

Considering shifts in visual perspective in the context of retrieval orientation is fruitful for better understanding how it interacts with emotional regulation. For example, active emotional regulation goals might bias how some individuals process retrieval cues in way that prioritizes adopting an own eyes or observer-like perspective during memory recall. This might explain why there is a higher frequency of observer-like perspectives reported in AMs in certain populations, such as post-traumatic stress disorder, who might avoid eliciting strong emotional responses during voluntary retrieval of AMs by emphasizing some features of memories over others (e.g., Berntsen et al., 2003; McIsaac and Eich, 2004). Another aspect of constantly adopting a certain visual perspective (and avoiding the other one) might be linked to implicit emotion regulation in which people modify their emotional experiences unintentionally (Mauss et al., 2007; Koole and Rothermund, 2011). One potential implication is whether the prioritization of an observer-like perspectives for some memories (e.g., traumatic events) could turn into habitual use of an emotional regulation strategy, without exerted control, over time (Gyurak et al., 2011; Braunstein et al., 2017) that leads memory details to be represented less salient in the long term (Koole and Rothermund, 2011). In this case, shifting to a novel visual perspective that is initially avoided might impair the functioning of the implicit emotional regulation and influence how memory details, including emotional aspects, are retrieved. Another critical question is how explicit (i.e., intentional) emotion regulation goals accompanying visual perspective shifts during retrieval might differentially influence the emotional aspects of AMs. Earlier theories have suggested that the time when the explicit emotion regulation goals are activated, following the presentation of an emotional stimulus, determines the effectiveness of the emotion regulation strategy. For example, Sheppes and Meiran (2007) showed that when people were instructed to employ cognitive reappraisal long after they started to watch emotional films, they had difficulty diminishing the negative affect triggered by the stimuli. In contrast, when cognitive reappraisal was initiated shortly after the presentation of emotional stimuli, it was more effective in down-regulating negative affect. Related to this idea, one question is how the temporal sequence of emotion regulation instructions and visual perspective cues could impact emotional experiences. For example, orienting retrieval with a visual perspective cue before setting the emotion regulation goal might help event details to be reconstructed earlier and facilitate the generation of the desired emotional response in contexts where the intentional emotion regulation goal may not be as effective, such as traumatic losses or extremely negative events.

The idea that own eyes and observer-like perspectives reflect different retrieval orientations could also explain reported differences in subjective and objective characteristics of memories due to visual perspective. If we assume that most memories are encoded from an own eyes perspective, then a retrieval orientation matching this viewpoint (i.e., own eyes) should be more effective than one that mismatches (i.e., observer). Prior research has primarily investigated how shifting from a dominant own eyes perspective to a novel observer-like perspective during retrieval influences remembering (e.g., Robinson and Swanson, 1993; Berntsen and Rubin, 2006; Sekiguchi and Nonaka, 2014; Vella and Moulds, 2014; Akhtar et al., 2017; St Jacques et al., 2017; King et al., 2022). Thus, changes in emotional and other recollective aspects due to shifting perspective could be explained by how retrieval orientation processes lead to a mismatch from encoding (Marcotti and St Jacques, 2018). This leads to the novel prediction that shifting from an observer-like to an own eyes perspective would be similarly ineffective in eliciting successful retrieval for memories that were initially encoded from an observer-like perspective, as this scenario would involve a similar mismatch in retrieval orientation and encoding processes. Prior research has further suggested that events involving self-conscious emotions are more likely to be encoded from a natural observer perspective (Nigro and Neisser, 1983; McCarroll, 2017, 2018), which leads to the intriguing possibility that adopting an observer-like perspective during retrieval of these events might better recapitulate the same processes engaged during encoding—thus, explaining why subjective emotionality and other recollective properties in such events may not change unless there is an explicit effort to regulate the experienced emotions. That is, the ineffectiveness of shifting from an observer-like to an own eyes perspective for these events can be relatable to retrieval orientation processes rather than self-evaluations (Sutin and Robins, 2008) or meaning-making (Libby and Eibach, 2011). Importantly, this does not entirely eliminate the idea that a particular visual perspective may cause people to evaluate themselves or appraise the memory content in alternative ways. Instead, our proposed theory suggests that focusing on the changes in basic retrieval processes due to perspective shift would give an essential understanding of why a presented visual perspective cue influences recollection even in the early stages of retrieval. An important step for future research will be to manipulate encoding of memories from an observer-like perspective (e.g., Iriye and St Jacques, 2020) in order to examine how orienting retrieval toward own eyes or observer-like perspectives prioritize different characteristics of memory retrieval. Shifting from a dominant perspective to a novel one, regardless of its direction, would be re-orienting retrieval processes to a viewpoint that does not recapitulate the original one, which biases the way that AMs are retrieved and specifically impacts emotional aspects of memory.

In conclusion, the flexible nature of memory enables people to adopt multiple visual perspectives during retrieval. The studies reviewed here demonstrate that updating the original visual perspective of AMs contributes to the reconstructive nature of retrieval and reshapes the subjective and objective measures of emotionality (St Jacques, 2019, 2022), thereby serving as an effective emotion regulation tactic (Webb et al., 2012; Wallace-Hadrill and Kamboj, 2016; Powers and LaBar, 2019). Here we also propose that own eyes and an observer-like perspectives are two distinct retrieval orientations that bias the way memories are retrieved. According to this theory, changes in the subjective sense of emotionality that emerged from visual perspective manipulation are the consequence of various factors related to both encoding and retrieval.

## Author contributions

SK and PLS conceived the ideas and wrote the manuscript. Both authors contributed to the article and approved the submitted version.
